# The Contribution of Sulfated Glycosaminoglycans to the Inflation Response of the Human Optic Nerve Head

**DOI:** 10.1167/iovs.18-23845

**Published:** 2018-06

**Authors:** Dan E. Midgett, Joan L. Jefferys, Harry A. Quigley, Thao D. Nguyen

**Affiliations:** 1 Department of Mechanical Engineering, The Johns Hopkins University, Baltimore, Maryland, United States; 2 Wilmer Ophthalmological Institute, School of Medicine, The Johns Hopkins University, Baltimore, Maryland, United States

**Keywords:** lamina cribrosa, optic nerve head, glycosaminoglycans, biomechanics, glaucoma

## Abstract

**Purpose:**

In this study, we measured the effect of the removal of sulfated glycosaminoglycans (sGAGs) on the pressure-induced strains of the human lamina cribrosa (LC).

**Methods:**

We applied an ex vivo inflation method to measure the three-dimensional (3D) deformation response of six human LCs to pressure, before and after the degradation of chondroitin and dermatan sulfates. The experiment used a laser-scanning microscope (LSM) to acquire the second harmonic generation (SHG) signal of the collagen structure in the LC. Digital volume correlation (DVC) was used to calculate the deformation in the LC after a change in pressure from 5 to 45 mm Hg.

**Results:**

The average strains between 5 and 45 mm Hg in the LC decreased significantly after sGAG degradation (*P ≤* 0.03), with the greatest change occurring in regions of previously high strain (*P ≤* 0.003) and the peripheral regions of the LC (*P ≤* 0.02). The stiffening effect was greater in the LC of middle-aged (42–49 years) donors compared with those of older (64–88 years) donors (*P* < 0.0001).

**Conclusions:**

The LC experienced less strain at the same pressures after most sGAGs were removed. These results suggest that the natural decrease in sGAGs within the LC with age may contribute to the stiffer inflation response of older LC to IOP. Likewise, the increase in the amount of sGAGs observed in the LC of glaucomatous eyes, may contribute to a more compliant LC, which may affect the susceptibility and progression of axon damage.

The axons of retinal ganglion cells exit the intraocular space through a porous connective tissue structure known as the lamina cribrosa (LC). The LC is formed by approximately 10 perforated plates, stacked on top of one another, which mechanically support the axons while helping to maintain the pressure gradient between the intraocular and extraocular spaces.^1^ Albon et al.^2,3^ showed that the LC is composed mainly of collagen (24%–47%) and elastin (7%–28%) by dry weight, with a strong dependence on age. By comparison, proteoglycans (PGs) make up a relatively small fraction of the LC (0.8%–2.3%) by dry weight. The PGs in the LC contain sulfated glycosaminoglycans (sGAGs) as a side chain, which occur in three varieties in the LC: chondroitin and dermatan sulfates, which are found near collagen fibrils, and heparan sulfate, which is found in the basal laminae of astrocytes and blood vessels.^4^

Numerous studies have shown that the LC remodels with age and glaucoma. The LC becomes stiffer with age,^5^ exhibits an increase in total collagen content from 24% to 47% between the ages of 0 and 89 years, a reduction in the amount of sGAGs of approximately 23 to 7 μg/mg between the ages of 0 and 93 years, and an increase in elastin content from 8% to 28% between the ages of 0 and 89 years by dry weight.^2^ Glaucoma is associated with increases in the amount of sGAGs.^6^ Tezel et al.^7^ showed that the amount of serum autoantibodies that bind to purified chondroitin and heparan sulfate glycosaminoglycans was 100% higher in patients with normal-tension glaucoma and 50% higher for patients with open-angle glaucoma. Glaucoma is also associated with a reduction in the density of types I and III collagen and increases in type IV collagen in the optic nerve head,^8–10^ and with morphological changes to elastin where the fibers appear more curled and disorganized.^11,12^

Modeling studies have suggested that the optic nerve head (ONH) stress and strain state is strongly influenced by IOP, and the mechanical properties of the LC and surrounding sclera.^13–17^ In particular, Sigal et al.^18^ found that the posterior bowing of the LC may be most sensitive to the elastic modulus of the LC. Recent advances in imaging and image processing methods have made possible detailed experimental measurements of the pressure-induced deformation response of the LC. Girard et al.^19,20^ used spectral-domain optical coherence tomography and digital volume correlation (DVC) to measure in vivo changes in the strains of the LC after pressure-lowering trabeculectomy. Sigal et al.^21^ used second harmonic generation (SHG) imaging of an ex vivo, human explant model and 2D digital image correlation to calculate strain changes in the LC structure due to increasing IOP. Coudrillier et al.^22^ used phase-contrast micro–computed tomography and DVC to study the correlation between IOP and strain in the LC and surrounding tissues in porcine eyes. In our recent work, we also demonstrated a method using SHG microscopy and DVC to calculate the 3D deformation resulting from an increase in IOP with micrometer-scale resolution.^5^ This work revealed a highly heterogeneous LC strain field with regions of large strains exceeding 10%. We showed that high strains occurred in localized regions that bulged more posteriorly in response to pressure. The strains were also larger in the peripheral rather than central region of the LC and were lowest in the nasal quadrant compared with the inferior, temporal, and superior quadrants. These findings are consistent with the topographical loss of axons in human glaucoma eyes, which tends to develop first in the superior and inferior poles, with corresponding defects in the visual field in areas subserved by these affected axons.^23,24^ Later observations by Jonas et al.^25^ confirmed that neuroretinal rim loss in glaucoma was greater in the superotemporal and inferotemporal zones of the ONH.

The sGAGs may influence LC mechanical behavior by influencing the hydration of the tissue and by modulating the interactions between collagen fibrils. The molecules attract and hold onto water through negative ionic charges^26^ and osmotic pressure.^27–29^ They also directly interact with collagen fibrils via electrostatic interaction^30–34^ and have been hypothesized to regulate collagen fibril spacing through hydration,^29,35,36^ GAG-to-GAG charge repulsion,^37^ and GAG-to-GAG anti-parallel interactions.^38^ The number of GAGs in the human LC is also known to change with age and glaucoma,^2,6,7^ thus characterizing the role of sGAGs on the biomechanics of the LC is essential for understanding the effect that changes in the amount and distribution of sGAGs with age and glaucoma may have on the development of axonal damage.

In this work, we applied the ex vivo inflation test with SHG imaging and DVC, developed previously,^5^ to investigate the contribution of sGAGs to the mechanical behavior of the human LC. Human donor sclerae were incubated in buffer for 4 hours and imaged before and after a change in IOP. The sclerae were then incubated in a solution containing chondroitinase ABC (ChABC), to digest the sulfated chondroitin and dermatan sGAGs,^39,40^ and re-imaged at the same pressures. DVC was then used to calculate the displacement fields and strains resulting from the change in IOP.

## Methods

### Glycosaminoglycan Quantification

The protocol for sGAG quantification was described previously in Murienne et al.^39,40^ The posterior sclera of two eyes, a left eye (LE) and right eye (RE) pair, were prepared as described in Murienne et al.^40^ and used to measure the effectiveness of sGAG removal by ChABC over a 4-hour incubation period. The posterior scleral cup from the RE was incubated for 4 hours at 37°C in a Trizma buffer of pH 8.0, containing no ChABC enzyme (buffer-treated), while the specimen excised from the LE was incubated for 4 hours at 37°C in a Trizma buffer of pH 8.0 containing ChABC enzyme (C2905; Sigma-Aldrich Corp., St. Louis, MO, USA) at 2 units mL*^−^*^1^ (enzyme-treated). ChABC is known to specifically degrade chondroitin and dermatan sulfates at pH 8.0 and 37°C.^39,40^ Square samples of size 3 × 3 mm were then cut out of the superior-nasal (SN), inferior-nasal (IN), inferior-temporal (IT), and superior-temporal (ST) quadrants of the scleral cups, at equal distances from each other and 5 mm from the center of the LC. An additional sample containing the entire LC was also cut out from each eye. Each sample was cut into two parts of size one-fourth and three-fourths, creating two samples from each area. Both the large and the small samples from each area were weighed after blotting dry on Whatman paper, using a precision balance (XP26DR; Mettler-Toledo LLC, Columbus, OH, USA). sGAG content per wet tissue weight was assessed in the larger sample using the Blyscan assay (Accurate Chemical and Scientific Corporation, Westbury, NY, USA) and the protocol described by Boubriak et al.^41^ The smaller samples were dehydrated at 60°C for 48 hours and weighed in the same manner as before. The sGAG content per dry tissue weight for each larger sample was then inferred from the ratio of the dry to wet weights measured for the smaller sample. The thickness at eight locations in the sclera before and after buffer and enzyme treatment was measured using a custom ultrasonic device, as described in Murienne et al.,^40^ for the LE and RE specimens (Supplementary Material S4; Supplementary Table S4).

### Specimen Preparation and Inflation Test

Seven human eyes from six donors (ages 42, 49, 61, 64, 79, and 88 years) were obtained from the National Disease Research Interchange and Eversight Eye Bank. Donor eyes were shipped on ice in wet gauze within 24 to 48 hours postmortem. The experimental group included eyes from four male and two female donors with a mean age of 63.8 *±* 17.4 years and no history of glaucoma (Table 1). We affirm that our research followed the tenets of the Declaration of Helsinki.

**Table 1:**
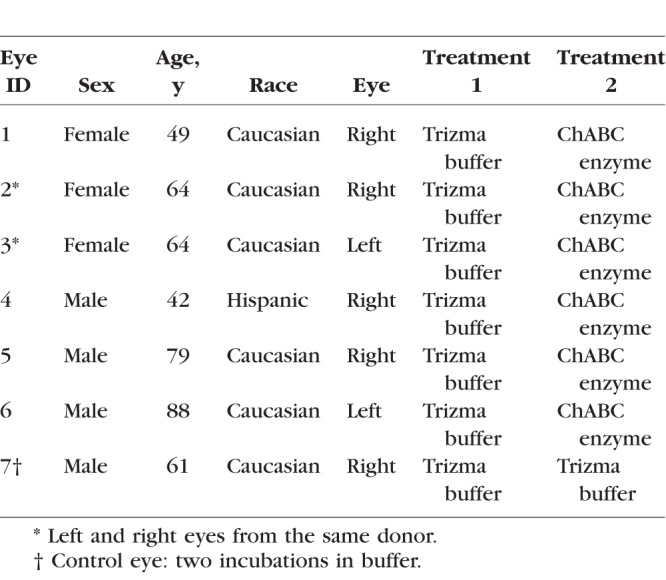
Human Donor Posterior Sclerae Subjected to Inflation Testing

**Figure 1:**
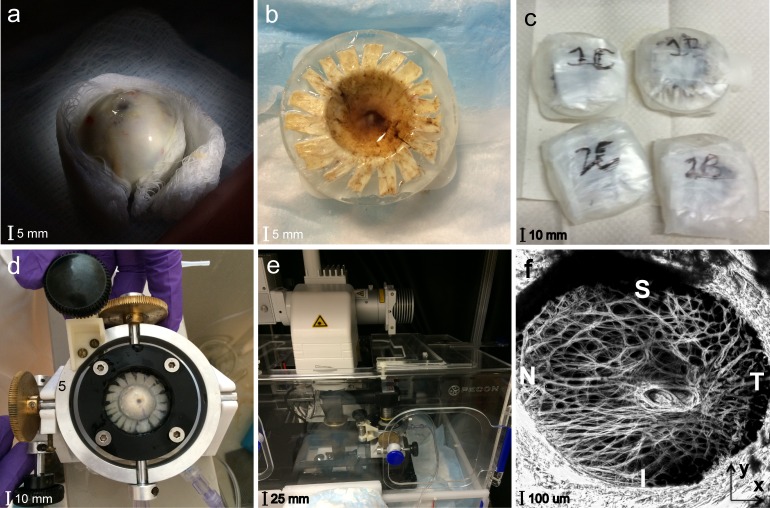
Experimental setup for the inflation test of the human LC showing the (a) ONH cut flush to the sclera; (b) ring-mounted posterior scleral cup; (c) posterior scleral samples incubating at 37^°^C; (d) custom inflation holder; (e) Zeiss LSM 710; and (f) maximum intensity projection, SHG image of the LC.

Posterior scleral specimens were prepared and imaged within 72 hours postmortem at 18°C as previously described.^5^ In brief, the optic nerve was cut flush with the sclera to expose the LC (Fig. 1a), then the cornea, anterior sclera, choroid, and retina were removed. The posterior scleral cup was glued to a polycarbonate ring, which was inverted and placed in a plastic dish (Fig. 1b). The plastic dish and inside of the scleral cup were then filled with a Trizma buffer, sealed in parafilm (Fig. 1c), and buffer-treated as described previously in Glycosaminoglycan Quantification to ensure that all samples were at a similar hydration before testing.^39^ After 4 hours, the posterior scleral specimen was removed from the buffer and mounted on a custom inflation holder (Fig. 1d). The extraocular surface was then immersed in PBS, and the posterior surface of the LC was aligned with a 10× 0.45 NA Apochromat water-immersion objective, which was inserted into the fluid column and inverted for overhead imaging with a Zeiss, laser-scanning microscope (LSM 710 NLO; Oberkochen, Germany). A coherent Chameleon Ultra II laser tuned to 780 nm in SHG imaging mode was used, with the signal collected from a 390- to 410-nm band pass filter (Fig. 1e). The pressure was set to 5, 10, and 45 mm Hg using an external water column, and each specimen was equilibrated for at least 25 minutes at each pressure before imaging. Because the LC experienced significant posterior deflection during this pressure increase, a distinct landmark in the LC structure was selected and imaged for reference at 5 mm Hg. At higher pressures, this landmark was relocated and the focal depth adjusted so as to minimize the imaging of dark featureless areas and reduce the imaging time. This focal adjustment was recorded and added back to the anterior-posterior displacement calculations. Two 2 × 2 tiled, duplicate z-stacks of the LC volume were captured at each pressure, starting at the lowest visible depth in the LC with images taken sequentially every 3 μm up to the posterior LC surface. This method was able to image through to a thickness of 150 to 300 μm from the posterior surface of the LC. To minimize errors from tissue creep during image acquisition, the tiled z-stacks were acquired with a short pixel dwell time of 5 μs and no line averaging. These settings produced a 3- to 6-minute imaging time for each tiled z-stack, with volume sizes and imaging time being constant for all z-stacks of the same eye. The X and Y directions within each image were aligned with the nasal-temporal (N-T) and inferior-superior (I-S) axes of the LC, respectively, and the out-of-plane or anterior-posterior direction was designated as Z (Fig. 1f).

After imaging, specimens were removed from the inflation chamber, inverted, and placed in a plastic dish. Six of the seven specimens were incubated in enzyme as described previously in Glycosaminoglycan Quantification. The seventh eye was again incubated in the buffer solution as a control. The specimens were then remounted in the custom inflation chamber and subjected to the same pressurization and imaging protocol as before.

### Displacement and Strain Calculations

The image post-processing, DVC method for displacement correlation, strain calculation, and error analyses are described in detail in Midgett et al.^5^ In brief, the Fast-Fourier Iterative DVC algorithm^42^ was used to analyze the z-stack volumes to calculate the 3D displacement field *U_X_*, *U_Y_*, and *U_Z_* between the baseline pressure of 5 mm Hg and the higher pressures of 10 and 45 mm Hg. The components of the Green-Lagrange strain tensor were calculated by fitting sixth-order polynomial functions to the components of the displacement field and evaluating the gradient. The magnitude of strain experienced by the tissue at each point varies depending on the direction considered. In this analysis, the normal strain (elongation) along to the N-T direction is denoted as *E_XX_*, the normal strain along the I-S direction is denoted as *E_YY_*, and the shear strain (angular distortion) within the N-T and I-S plane is denoted as *E_XY_* . These strains were calculated every four pixels (10 μm) in the X-Y plane within the images, and every two slices (6 μm) in the Z direction, resulting in a high-resolution, 3D strain field within the LC for analysis. These three strain measures were also used to calculate the maximum principal strain, denoted as *E_max_*, which is the magnitude of the normal strain within the NT-IS plane at which it is maximum, and the maximum shear strain, denoted as Γ*_max_*, which is the magnitude of the shear strain along the orientation at which it is maximum (Supplementary Material S1). All five strain fields for the LC, both before and after sGAG degradation, were averaged through Z at each X and Y point, and then the strain fields in X and Y were compared. We estimated the baseline errors intrinsic to the DVC method and caused by specimen creep during image acquisition by correlating two z-stacks acquired back-to-back at 45 mm Hg under nominally identical conditions (Supplementary Material S2.1). In addition, we estimated the displacement and strain errors by numerically applying a translation of 10 pixels in the X and Y directions, a translation of 3 pixels (slices) in Z direction, a 2% uniform stretch along the X and Y directions, and a 5% uniform compression in the Z direction to one of the duplicate z-stacks and correlated it with the undeformed z-stack (Supplementary Material S2.2).

### Regional Division

**Figure 2:**
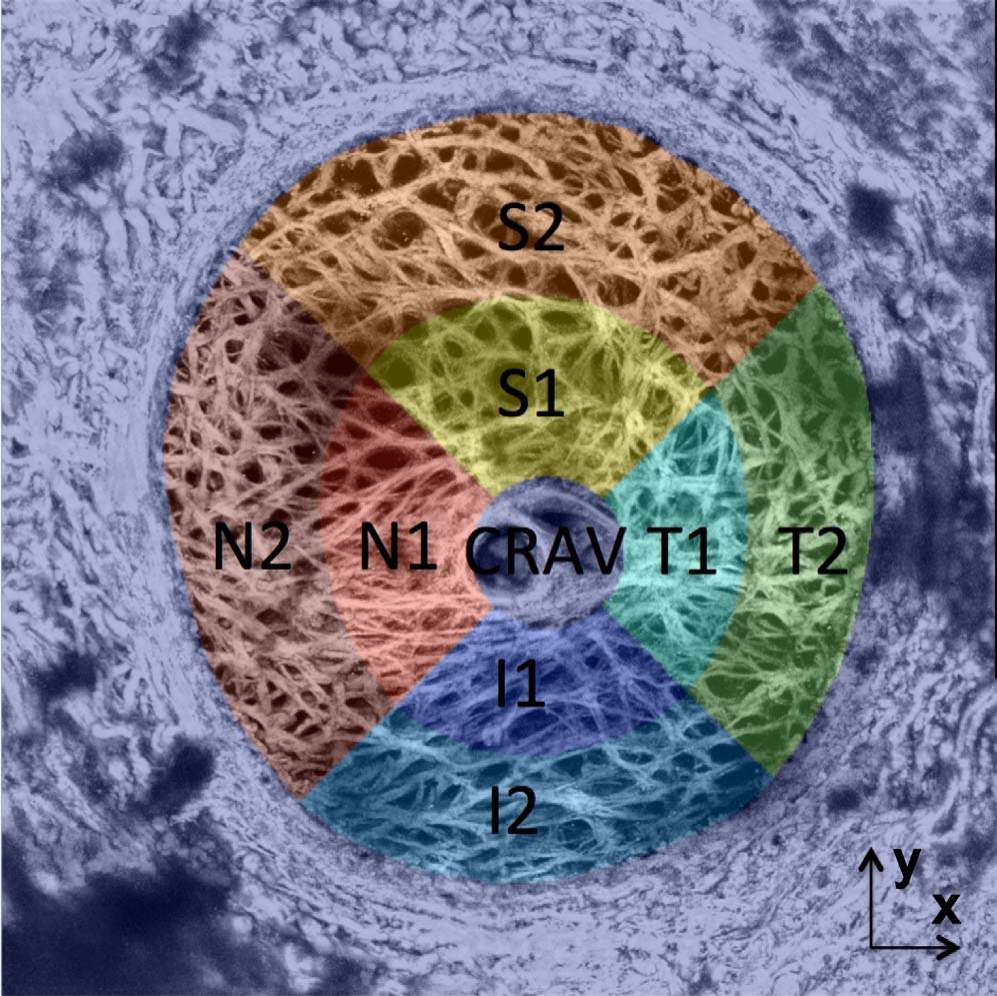
The segmentation of the LC from the PPS and the eight LC regional divisions for specimen 2.

The LC was divided into eight regions to analyze for regional variations in the LC strains. To define the regions, the shape of the LC opening was calculated by importing the z-stacks at 5 mm Hg into FIJI^43^ (National Institutes of Health, Bethesda, MD, USA) and converting them to a maximum intensity z-projection. Points on these projections were picked manually that separated the LC from the peripapillary sclera (PPS), which is denser in collagen and appears as a bright oversaturated region in the SHG images. An ellipse was fit to the points using the MATLAB function *fit_ellipse*,^44^ and used to segment the displacement field corresponding to the LC from the PPS. The LC was divided into eight regions (Fig. 2) centered about the central retinal artery and vein (CRAV). The CRAV region was defined as the cylindrical region within *r* < 200 μm of the CRAV center point. The center point of the CRAV was manually picked by marking a point on the CRAV in the z-projected, reference image in FIJI, and then overlaying the circle of radius 200 μm to check if it completely encircled the CRAV with the artery and vein centered. The central and peripheral regions of the LC were defined by calculating the mid-radial distance between the LC opening and the CRAV circle. The central and peripheral regions were further divided into quadrants, superior (S), inferior (I), temporal (T), and nasal (N), using 45° and 135° bisectors centered about the CRAV.

### Statistical Analysis

We first investigated the average strain magnitude of the strain fields for each specimen (*n* = 6). The Wilcoxon signed rank test was used to compare these outcomes before and after enzyme treatment. Next, the change in the strain fields was investigated regionally, by treating the outcome measures from each of the eight regions of the LC as repeat measurements. Generalized Estimating Equation (GEE) linear models were used to determine: (1) the change in average strain magnitude after enzyme treatment, (2) the effect of the original average strain magnitude on the strain decrease after enzyme treatment, (3) the effect of central or peripheral regional location within the LC on the strain decrease after enzyme treatment, and (4) the effect of age on the strain decrease after enzyme treatment. For specimen 6, the inferior and peripheral temporal regions were mostly dark and failed to correlate (Supplementary Fig. S4), thus these regions were omitted from analysis resulting in 45 regional strain measures (*n* = 45).

Because only one human contributed data from both eyes, it was not possible to account for correlations between eyes in the analysis; thus, for all tests, the data from all six eyes were assumed to be independent. The GEE linear models take into account correlations in outcome among the regional measurements for a single eye. Measurements from the eight regions were assumed to have a compound symmetry correlation structure, in which the measurements from any two regions have the same correlation. Comparisons were considered significant if the *P* value was less than 0.05. Statistical analyses were performed using the software SAS (version 9.2; SAS Institute, Inc., Cary, NC, USA).

Average absolute strain, or average strain magnitude, was chosen for the comparison of *E_XX_* and *E_YY_* before and after enzyme treatment, rather than a simple average. The pressure-induced strain fields in the LC are predominantly tensile; however, compressive regions of strain also exist.^5^ If compressive regions of strain were averaged with nearby positive regions, the area-averaged strain would appear low even though the area actually had a high magnitude of strain. For this reason, we found average strain magnitude to be a better strain outcome for comparison.

### Measurement of Thickness Changes After sGAG Removal

Removing sGAGs can alter the spacing between the collagen fibrils and the hydration of the LC tissue. We measured the thickness change after sGAG removal to determine whether changes in the LC strains after ChABC treatment can be attributed to changes in the LC thickness. DVC was used to measure the shape change of SHG images acquired at 5 mm Hg before and after incubation in ChABC. The buffer-treated images at 5 mm Hg were used as the reference volume and the enzyme-treated images at 5 mm Hg were used as the deformed volume. This correlation calculated how different points in the LC volume moved anteriorly or posteriorly at 5 mm Hg after incubation in ChABC. To determine the thickness change at each X and Y point in the imaged, posterior LC volume, the average change in Z position at the anterior-most portion of the imaged LC volume was subtracted from the average change in Z position in the posterior-most portion of the imaged LC at each X and Y point. This yielded a thickness change map in X and Y. This field was then averaged to obtain the average change in thickness across the LC.

## Results

### sGAG Quantification

We measured a smaller sGAG content per dry weight (μg/mg) in the enzyme-treated samples compared with the buffer-treated samples using the Blyscan assay (see Glycosaminoglycan Quantification) for all regions of the sclera and the LC (Table 2). The sGAG content of the enzyme-treated LC sample was 14% that of the buffer-treated sample. In the sclera, the sGAG content of the enzyme-treated samples varied between 4% and 8% of that measured for the buffer-treated samples.

**Table 2:**
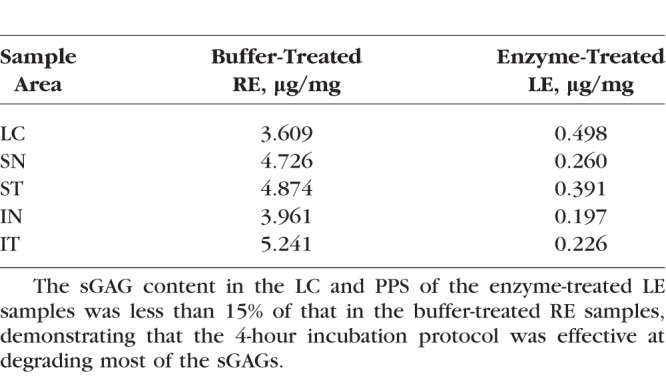
The sGAG Content Per Dry Weight in PPS and LC Samples

### Thickness Changes

The thickness of the LC for all specimens increased by 1 μm or less in five of the six specimens after sGAG degradation and decreased by 0.07 μm in one specimen (Table 3). The measured thickness changes were all smaller than the average DVC correlation error for the displacement component *U_Z_* in the thickness direction. Although thickness changes were very small in most of the imaged volume of the LC (Supplementary Figs. S1a–f), some localized regions showed thickness changes greater than the spacing between z-slices (3 μm). These regions were often closer to the periphery, but their location was not consistent between specimens.

**Table 3:**
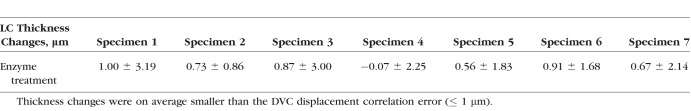
The Average Thickness Change Over the Imaged Volume of the LC After Enzyme Treatment

### Changes in LC Strains

**Figure 3:**
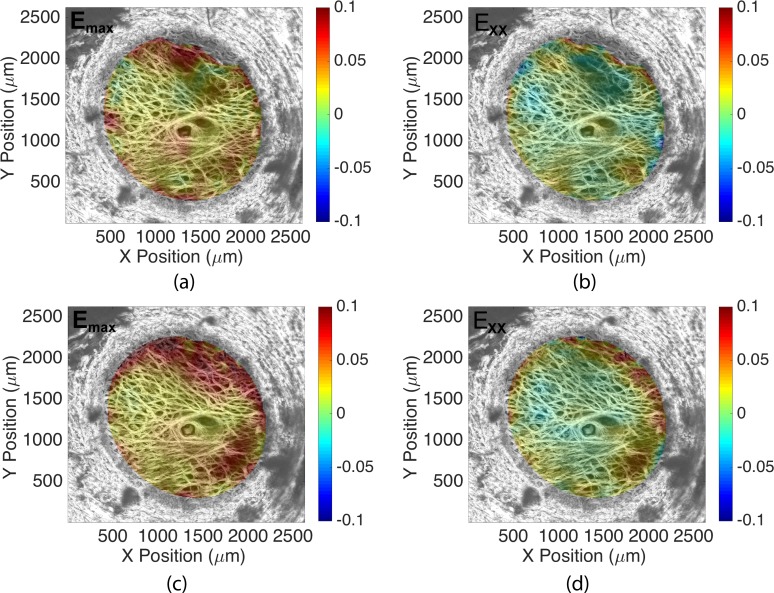
The change in the strain response of specimen 7 from 5 to 45 mm Hg after two incubations in buffer: (a) E_max_ after first buffer treatment, (b) E_XX_ after first buffer treatment, (c) E_max_ after second buffer treatment, and (d) E_XX_ after second buffer treatment. Both strain measures increased after the second buffer treatment.

Contour plots of the displacement fields, *U_X_*, *U_Y_*, *U_Z_*, the deflection in *Z*, and the in-plane strain components *E_XX_*, *E_YY_*, *E_XY_*, *E_max_*, and Γ*_max_* are shown for all specimens for an inflation from 5 to 45 mm Hg after incubation in buffer and incubation in enzyme in Supplementary Material S5. Specimen 7 was subjected to two sequential buffer treatments, once before each imaging session, to determine the effect of incubation in buffer alone on the pressure-induced strains. All strain outcomes generally increased after the second buffer treatment compared with the strains after only one buffer treatment (Fig. 3). On average, strain magnitudes for *E_XX_* increased by 0.0040, *E_YY_* increased by 0.0083, *E_max_* increased by 0.010, and Γ*_max_* increased by 0.0022 for inflation from 5 to 45 mm Hg. We used this comparison as a control for the enzyme treatment, which added ChABC enzyme to the buffer in the second inflation test.

When the second incubation included ChABC enzyme, the strains in the LC decreased rather than increasing, as they had with two buffer treatments (Fig. 4; Supplementary Figs. S5–S18). After sGAG degradation, the average strain magnitudes for *E_YY_*, *E_max_*, and Γ*_max_* decreased by 0.0063, 0.0081, and 0.0036 (*P* = 0.03, *n* = 6, for 5–45 mm Hg; Table 4). The average magnitude of *E_XX_* also decreased by 0.0041, although the result had a lower statistical significance (*P* = 0.06, *n* = 6, for 5–45 mm Hg; Table 4).

**Figure 4:**
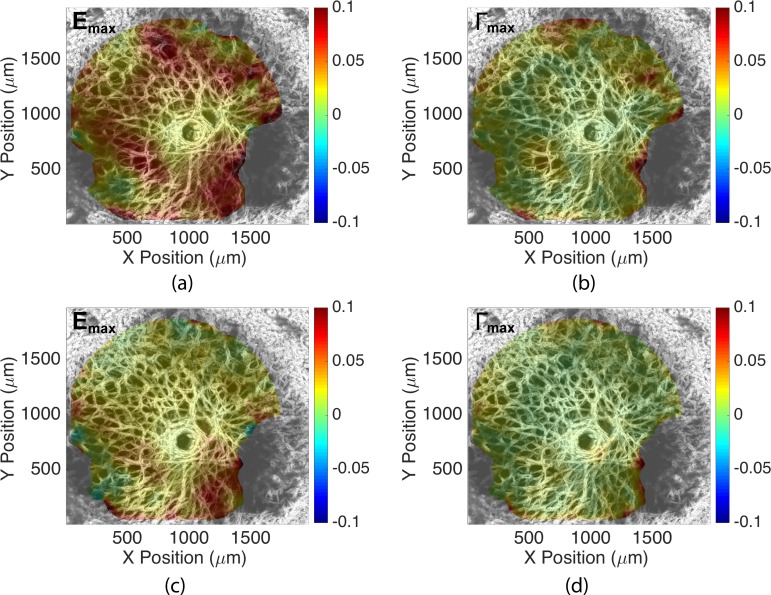
The change in the strain response of specimen 1 from 5 to 45 mm Hg after sGAG degradation: (a) E_max_ after buffer treatment, (b) Γ_max_ after buffer treatment, (c) E_max_ after enzyme treatment, and (d) Γ_max_ after enzyme treatment. Both strain measures decreased after enzyme treatment.

**Table 4:**
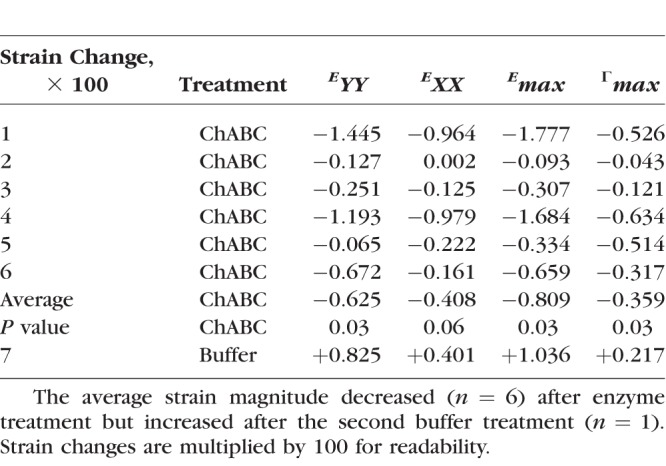
The Change in the Average Magnitude of the Specimen-Averaged LC Strains Between 5 and 45 mm Hg After Enzyme Treatment or After the Second Buffer Treatment

**Figure 5:**
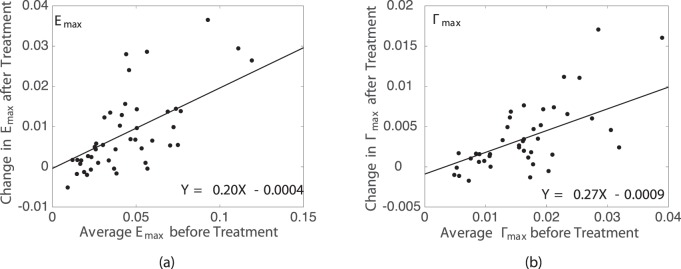
Correlation between the regionally averaged strain magnitudes at 45 mm Hg before treatment and the decrease in strain magnitude after treatment for (a) E_max_ and (b) Γ_max._

**Figure 6:**
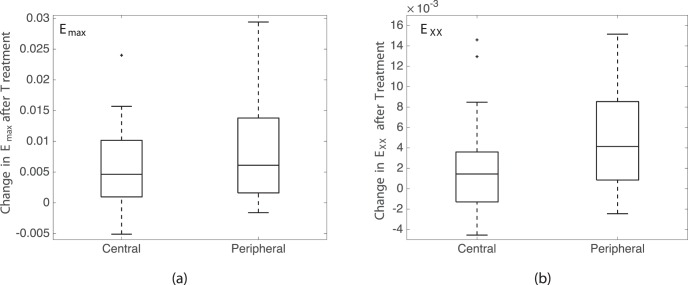
Comparison of the decrease in the regionally averaged strain magnitude at 45 mm Hg after sGAG degradation for central and peripheral LC regions (P = 0.02) for (a) E_max_ and (b) E_XX_.

We next calculated the average magnitude of *E_XX_*, *E_YY_*, *E_max_*, and Γ*_max_* for the eight regions in the LC for each eye before and after enzyme treatment, and applied a GEE linear model to analyze for strain changes after sGAG degradation, variations with region, and variations with age, accounting for repeated measures in each specimen. The decrease in strain outcome was found to be highly statistically significant (*P ≤* 0.01; Table 5) for *E_YY_*, *E_max_*, and Γ*_max_* and also significant for *E_XX_* (*P* = 0.03; Table 5). The decrease in the strain magnitude for each region after sGAG removal was also strongly correlated with the average strain magnitude before sGAG removal in that region (*P ≤* 0.003 for 5–45 mm Hg; Fig. 5; Table 6) for all strain measures but *E_XX_* at 45 mm Hg. All strain measures exhibited this trend for the lower pressure change of 5 to 10 mm Hg (*P ≤* 0.01 for 5–10 mm Hg; Supplementary Material S4). Regions with high average strain magnitudes before sGAG degradation exhibited larger strain decreases, whereas regions with low average strain magnitudes before sGAG degradation either did not show an appreciable change or experienced a slight increase in strain magnitude. The strain outcomes *E_XX_*, *E_max_*, and Γ*_max_* also experienced larger decreases after sGAG degradation in the peripheral compared with the central regions (*P ≤* 0.02 for 5–45 mm Hg; Fig. 6; Table 7). We did not find significant differences in strain changes between quadrants.

**Table 5:**
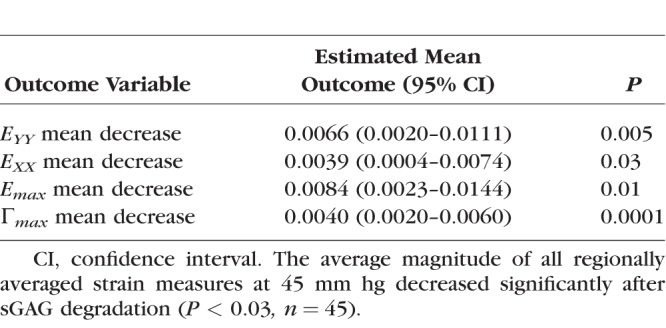
Strain Decreased Significantly After sGAG Degradation

**Table 6:**
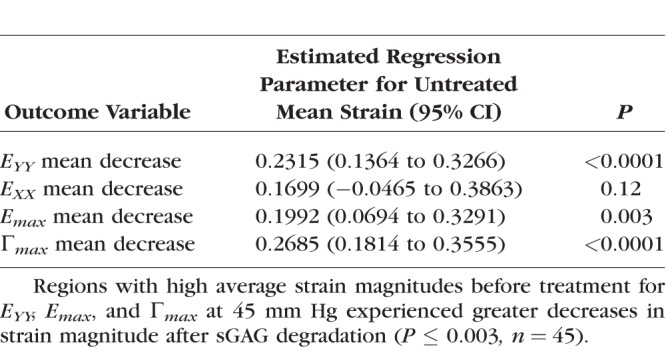
Strain Decreased Significantly More After sGAG Degradation in Regions With Previously High Strain

**Table 7:**
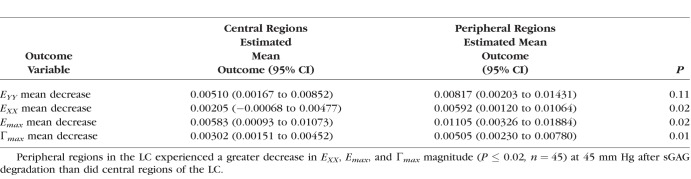
Strain Decreased Significantly More After sGAG Degradation in Periphereal LC Regions

**Figure 7:**
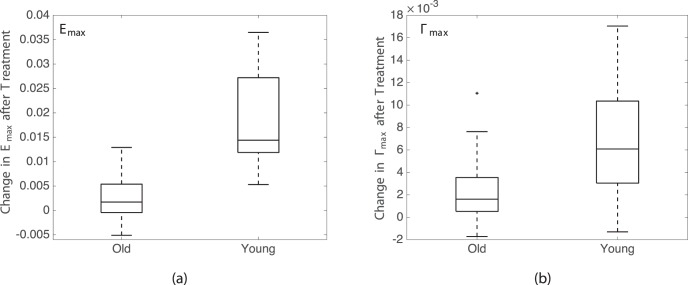
Comparison of the decrease in the strain magnitudes at 45 mm Hg after sGAG degradation in the LC of middle-aged and older-aged donor eyes: (a) E_max_ and (b) Γ_max_.

The six specimens came from donors who fell into two main age groups: middle-aged (42, 49) and older age (64, 79, 88), with a gap of 15 years between groups. Specimens from the older age group exhibited a statistically significant smaller decrease in all strain magnitudes after sGAG removal (*P* < 0.0001 for 5–45 mm Hg; Fig. 7; Table 8).

**Table 8:**
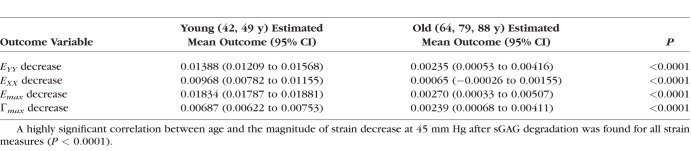
Strain Decreased Significantly More After sGAG Degradation in Younger Eyes

A similar decrease in the strain magnitude, and similar age and regional effects were found after enzyme treatment for the smaller pressure increase from 5 to 10 mm Hg, but with a lower statistical significance (Supplementary Material S4).

## Discussion

We measured changes to the thickness and pressure-induced strain response of the human LC after enzymatic degradation of sGAGs by ChABC for 4 hours. A Blyscan assay was used to measure the sGAG content of a buffer-treated LE and enzyme-treated RE to determine the effectiveness of sGAG degradation (see Glycosaminoglycan Quantification). The sGAG content of the enzyme-treated LC sample was 14% that of the buffer-treated specimen. For the four PPS samples, the enzyme-treated eye had only 4% to 8% of the sGAG content of the buffer-treated eye. These results demonstrate that a 4-hour incubation protocol in ChABC was sufficient to degrade most sGAGs. The remaining percentage could represent sGAGs not sensitive to ChABC, such as heparan sulfates, or partially degraded or incompletely removed dermatan and chondroitin sulfates. The buffer-treated sGAG content for the PPS samples calculated here was similar to that reported in previous studies.^40,41^

Degrading sGAGs consistently altered the strain response of the LC to pressure without appreciably changing the thickness of the imaged volume of the LC. We developed a method using DVC analysis of the enzyme-treated and buffer-treated volumes to measure the spatially varying thickness changes in the LC caused by sGAG removal. On average, the thickness changed by less than 1 μm over the 200 to 300 μm imaged volume. Thickness changes exhibited some regional variation in the LC, but regions with large changes in strain did not correspond consistently to areas with significant changes in thickness (Supplementary Figs. S1a–f). For this reason, we do not believe that thickness changes in the LC contributed significantly to the strain changes seen after sGAG removal. In contrast, Murienne et al.^40^ found that after sGAG degradation using an 18-hour incubation protocol in ChABC, the sclera thinned by 10%. We demonstrated a similar thinning in the sclera of 10% on average, for an eye after incubation in ChABC for 4 hours (Supplementary Table S4).

All strain outcomes, both the normal strain components along the nasal-temporal and inferior-superior directions, the maximum principal strain, and the maximum shear strain, decreased in all eight regions of the LC after sGAG degradation. We found the opposite for the two-times buffer-treated control specimen, in which LC strain outcomes all increased after the second buffer treatment. After enzyme treatment, for an inflation from 5 to 45 mm Hg, the strain magnitudes for *E_XX_*, *E_YY_*, *E_max_*, and Γ*_max_* for the six specimens decreased on average by 16.1%, 20.6%, 18.9%, and 19.8%. The strain reductions were statistically significant when comparing the specimen-averaged strain magnitudes, and highly statistically significant when comparing regionally averaged strain magnitudes. Murienne et al.^40^ previously reported that sGAG removal increased the stiffness of the measured stress-strain response and decreased the specimen-averaged meridional and circumferential strains of the human sclera by 10% and 0%, respectively. Assuming that the sclera was also stiffened in this study and noting that the thickness and diameter of the ONH did not change appreciably, the decrease in the LC strains is likely caused by a similar increase in the LC stiffness. Otherwise sGAG removal would cause the sclera to become even stiffer compared with the LC, which would have caused greater LC bowing and greater LC strain. On the contrary, the strain reduction was shown to be even greater in the LC in this study, compared with the reduction in scleral strain measured by Murienne et al.^40^ This suggests that sGAG removal increased the stiffness of the LC more than the stiffness of the sclera.

The reduction in the strains experienced by the LC due to inflation was found to follow predictable patterns. Regions with previously high strain magnitudes experienced the greatest reduction in strain, whereas those with low strain magnitudes exhibited little or no changes after sGAG removal. We also noted that the specimens with the largest reductions in strain (specimens 1, 4, and 6) appeared to exhibit the greatest reduction in the posterior bowing of the LC. Areas that bulged posteriorly at 45 mm Hg were reduced after sGAG degradation, which seemed to result in the greater strain reduction in these areas (Supplementary Figs. S5–S18). The stiffening effect of sGAG removal also differed by age and region. The nasal-temporal strain component *E_XX_*, the maximum principal strain *E_max_*, and the maximum shear strain Γ*_max_* experienced greater reduction in the peripheral regions than in the central regions of the LC. All strain components also had significantly larger reductions in strain within eyes from the younger age group (42 and 49 years) than from the older age group (64, 79, and 88 years). The smaller effect may have been measured for the older age group because the amount of sGAGs decreases in the LC with age.^2^ The decrease in sGAG content may also contribute to the age-related stiffening in the pressure-induced strain response of the LC measured in our prior study^5^ in addition to the increase in the collagen content^3^ and the expected increase in collagen crosslinking with age. Likewise, the increases in the amount of sGAGs in the LC of glaucomatous eyes^6,7^ may lead to a more compliant LC, which may affect the progression of glaucomatous axon damage. The larger effect of sGAG degradation in the peripheral LC region may be caused by regional variation in sGAG content, which may or may not be associated with the lower connective tissue density of the peripheral LC.^1,45^ The regional variation in strain reduction may also reflect changes in the mechanical interaction of the PPS and LC with sGAG removal, as the LC experienced a greater percentage decrease in strain after sGAG removal than that measured for the PPS by Murienne et al.^40^

There were several limitations to this study. Although the DVC method calculates high-resolution displacement and strain fields, there is a resolution limit to the method. For this study, we estimated this resolution limit by numerically applying a triaxial strain field and displacement and calculating the displacement and strain error for the 5 mm Hg reference volume of each eye. The average resolution for the in-plane strain measures *E_XX_*, *E_YY_*, and *E_XY_* was calculated as 0.0020, 0.0014, and 0.0013. Specimens from the youngest donors, specimen 1 and specimen 4, experienced strain changes an order of magnitude greater than this resolution after sGAG digestion, and specimens 3, 5, 6, and 7 experienced strain changes that were larger, but of the same order of magnitude as the DVC strain error. However, specimen 2 experienced small strain changes that were near the DVC strain error. Tissues were also tested postmortem. Specimens were kept chilled at 2°C for up to 48 hours before the inflation experiments, but the connective tissue structure may have been altered during storage and by the subsequent incubation process at 37°C. This is supported by the control specimen, which was incubated in buffer twice and the second incubation in buffer caused an appreciable increase in strains. Another limitation is that only one control eye was considered in this experiment, and the effect of incubating in buffer twice may vary by specimen, age, and time postmortem. Although the specimens were mainly from Caucasian donors, one eye was from a Hispanic donor. There may be racioethnic differences in the structure, biomechanics, and sGAG content of the LC. In addition to this, our study contained an LE and RE from the same donor. The inflation response of both eyes exhibited a lower than average change in strain. However, the correlation between eyes of the same donor and racioethnic differences were not factored into the statistical models because of the small sample size. We also were unable to image and accurately measure the strains and thickness changes in the PPS along with LC because the duration of the test would have been intractable. A study considering strain changes in both tissues at the same time would shed more light on the phenomenon of strain changes after sGAG degradation in the LC and sclera. The regional variation in the strain changes after sGAG removal may have been caused by regional variation in the sGAG content of the LC; however, we were unable to measure the latter because of the small size of the LC. Dividing the LC into four samples caused the sGAG content of the sample to fall below the detectable limit. Although we do not expect ChABC treatment to alter the elastin and collagen content of the LC, we did not directly verify this assumption; however, we did not observe gross changes in the SHG images (for collagen) and two photon fluorescence (TPF) images (for elastin) after sGAG degradation. The shape of the LC beams appeared the same before and after digestion.

## Conclusions

The strain response of the human LC to a pressure change from 5 to 10 and 5 to 45 mm Hg was compared before and after sGAG degradation. After sGAG degradation, the following changes were observed:

1. The LC experienced smaller strains in response to pressure.
2. Strains decreased more in areas of previously high average strain.
3. The strains in the nasal-temporal direction (*E_XX_*), the maximum normal strains (*E_max_*), and the maximum shear strains (Γ*_max_*) decreased more on the periphery near the PPS.
4. Older eyes exhibited a significantly smaller stiffening effect.
5. The average thickness changes of the LC after sGAG removal were smaller than the DVC displacement correlation errors.

sGAGs appear to play a significant role in the response of the LC to pressure, with the LC experiencing less strain after most sGAGs were removed. These findings may have important implications in understanding the biomechanical effects of changes in sGAGs with age,^2^ myopia,^46^ and glaucoma.^6,7^
